# The Differential Diagnosis between Malarial and Typhoid Fevers*Read before the annual meeting of the Michigan State Medical Society.

**Published:** 1897-10

**Authors:** J. F. Jenkins

**Affiliations:** Tecumseh, Mich.


### THE DIFFERENTIAL DIAGNOSIS BETWEEN MALARIAL AND TYPHOID FEVERS.*

BY J. F. JENKINS, M. D., TECUMSEH, MICH.

The difficulties in making a diagnosis between malarial fever and typhoid fever are in many instances very great, from the fact that typhoid fever presents itself under so many and varied conditions, the type at one time being very marked, at another time assuming many of the phases of malarial fever. Sporadic cases occurring in country districts and very generally in villages are not infrequently diagnosed malarial fever, and doubtless the deaths reported to local boards of health under the head of malarial fever are usually due to typhoid fever.

Dr. Osler states in a paper recently read by him, that "North of Mason and Dixon's line physicians are prone to diagnose malaria for other diseases; south of the line they are more prone to diagnose other diseases for malaria; in both regions it is a source of greater error than any other affection." There are many localities in this State, as well as other States north of Mason and Dixon's line, where malarial fever still prevails, especially during the autumnal months, and it is in these sections where it is so frequently found a difficult question to determine whether the patient has malarial or typhoid fever.

In each and every case the source of infection should be thoroughly investigated, and in many instances such an investigation will give material aid in arriving at a correct diagnosis.

It is seldom that typhoid fever commences with a pronounced chill, while malarial fever is usually ushered in with a chill, more or less severe, followed by high fever; sometimes typhoid fever commences with a distinct chill, that is, with a shaking chill, but the temperature is not so abrupt, never reaching 103 to 105 degrees, as it usually does in malarial fevers within the first twenty-four or forty-eight hours. The development of typhoid fever is in the large majority of cases slow and insiduous; there is a feeling of weariness, general malaise, some fever which increases day by day, and under these conditions quinine may be administered in antiperiodic doses which may lessen the temperature, but, like the annual overflow of the Nile, it is certain to again make its appearance. In malaria, epistaxis is rare, while in typhoid fever in its incipient stage, it is frequently present, especially among the young, but seldom occurring in patients after the age of fifty. In malarial fever the pulse rate will not be very much increased by taking the patient suddenly from bed and placing him in the upright position, while in the incipient stage of en-

* Read before the annual meeting of the Michigan State Medical Society.

teric fever the pulse rate is greatly increased by such a change of position. In the early stage of typhoid fever this alteration of position will frequently assist in determining the fever.

Where malarial fever prevails, and it is a question whether or not the infection is the product of Eberth bacilli or the hematozoa of malaria, ten grains of quinine given every three or four hours until thirty grains are administered, and this amount repeated every twenty-four hours for two or three days will materially assist in differentiating the disease. The infection of malaria will be destroyed by quinine, while in typhoid fever it may reduce the temperature, but it will soon reappear. Among the lagoons of northern India, more than a quarter of a century ago, the writer followed out a similar plan to the one above mentioned, and frequently found that instead of malarial infection, the only factor in the case was the infection of typhoid fever. These fevers commonly had all the characteristics of typhoid fever, excepting that they were invariably accompanied with profuse perspiration. The local physicians termed them "slow fevers," and finally they assume the name of typho-malarial fevers, but post-mortem examinations, whenever made, revealed the pathologic lesions of typhoid fever.

The importance of making an early diagnosis in typhoid fever is generally admitted, because the *hygienic* and *dietic management* of the *disease* is *absolutely* of *more importance than internal medication*; the lesion in the alimentary canal often leading to fatal results, even in apparently the mildest forms of enteric fever.

Within the past few years the microscope has made us familiar with the typhoid bacillus, and bacteriologists have made us acquainted with its natural history, its growth, its favorite habitate for development, how it behaves within and without the body, its most favorable conditions for propagation, and how to avoid and destroy the bacilli *without* the body. These facts are still leading us to more certain methods of diagnosis, and instead of depending upon symptoms we may look forward to methods which will be more definite in making out a differential diagnosis of fevers.

Ehrlich's test, or what is termed the diazo-reaction, appears to have a limited value. This test, which it is not necessary to describe here, responds to after the first week of typhoid fever; it may be used to determine whether the infection is due to the Eberth bacillus or the hematozoa of malaria.

Another means of diagnostic import in suspected cases of typhoid fever is to examine the stools for the Eberth bacillus and when found it is evident the patient has enteric fever.

The serum diagnosis of typhoid fever has been undergoing investigation during the past year, and its practical usefulness is still an unsettled question. The difficulty in Johnston's or Widal's test, as well as others above mentioned, is that it requires an expert, and hence they are almost useless in general practice.

The discovery of the hematozoa of malaria by Laveran is at least next in importance to the discovery of the tubercle bacillus by Koch. It has given the medical profession an intelligent concept of malarial fever in its various manifestations, and the literature on this subject has already been cleared of an immense amount of rubbish which has enveloped the medical practice of the past.

The combined infection of Eberth's bacillus and the hematozoa of malaria seldom occurs, excepting that it may still linger in the mind of some medical hayseed, who, Rip Van Winkle like, continues to hold fast to the opinion that malarial fever develops into typhoid fever, or is in some way dove-tailed into it. On this question permit me to quote Doctor Osler, who states that, "Among the one thousand cases of malaria and the five hundred cases of typhoid fever which have been at my clinic, almost every one of which has had a blood examination, there has been but one doubtful case of double infection."

Double infection so infrequently occurs that it will seldom enter as a factor in the differential diagnosis between malarial and typhoid fevers.—*Charlotte Medical Journal.*

